# Crystal structure and Hirshfeld surface analysis of dimethyl 3-methyl-8-{[4-(tri­fluoro­meth­yl)phen­yl]sulfon­yl}-7,8-di­hydro-4*H*-4,6a-ep­oxy­benzo[*b*]naphtho­[1,8-*de*]azepine-5,6-di­carboxyl­ate

**DOI:** 10.1107/S2056989025004426

**Published:** 2025-05-23

**Authors:** Gleb M. Burkin, Selbi Annadurdyyeva, Alexandra G. Kutasevich, Narmina A. Guliyeva, Khudayar I. Hasanov, Mehmet Akkurt, Gizachew Mulugeta Manahelohe

**Affiliations:** a RUDN University, 6 Miklukho-Maklaya St., Moscow 117198, Russian Federation; b Department of Chemical Engineering, Baku Engineering University, Hasan Aliyev, str. 120, Baku, Absheron AZ0101, Azerbaijan; c Azerbaijan Medical University, Scientific Research Centre (SRC), A. Kasumzade St. 14. AZ 1022, Baku, Azerbaijan; d Department of Physics, Faculty of Sciences, Erciyes University, 38039 Kayseri, Türkiye; e Department of Chemistry, University of Gondar, PO Box 196, Gondar, Ethiopia; Institute of Chemistry, Chinese Academy of Sciences

**Keywords:** crystal structure, disorder, acyl­ation, furan, sulfonamide, 4+2 cycloaddition, weak inter­actions, Hirshfeld surface analysis

## Abstract

The mol­ecular conformation of the title compound, C_29_H_22_F_3_NO_7_S, is stable due to the intra­molecular C—H⋯O hydrogen bonds. The central seven-membered ring adopts a distorted chair form. In the 7-oxabi­cyclo­[2.2.1]hepta-2,5-diene unit, the five-membered rings adopt envelope conformations. In the crystal, the mol­ecules are linked by C—H⋯O and C—H⋯F inter­actions, forming sheets parallel to the (002) plane. Additionally, S—O⋯π and π–π inter­actions [centroid-to-centroid distance = 3.6159 (7) Å] connect the mol­ecules along the *a-*axis direction. van der Waals inter­actions between the mol­ecular sheets reinforce the mol­ecular packing. A Hirshfeld surface analysis was conducted to visualize the various inter­molecular inter­actions, indicating that the largest contribution to the surface contacts is from H⋯H inter­actions (37.3%), followed by O⋯H/H⋯O (24.1%), F⋯H/H⋯F (19.0%), and C⋯H/H⋯C (10.3%) inter­actions.

## Abstract 2

In the crystal, mol­ecules are linked by C—H⋯O inter­actions and C—H⋯F inter­actions to form sheets parallel to the (002) plane. In addition, S—O⋯π and π–π inter­actions link mol­ecules along the *a-*axis direction. van der Waals inter­actions between mol­ecular sheets consolidate the packing.

## 1. Chemical context

**Figure 1:**
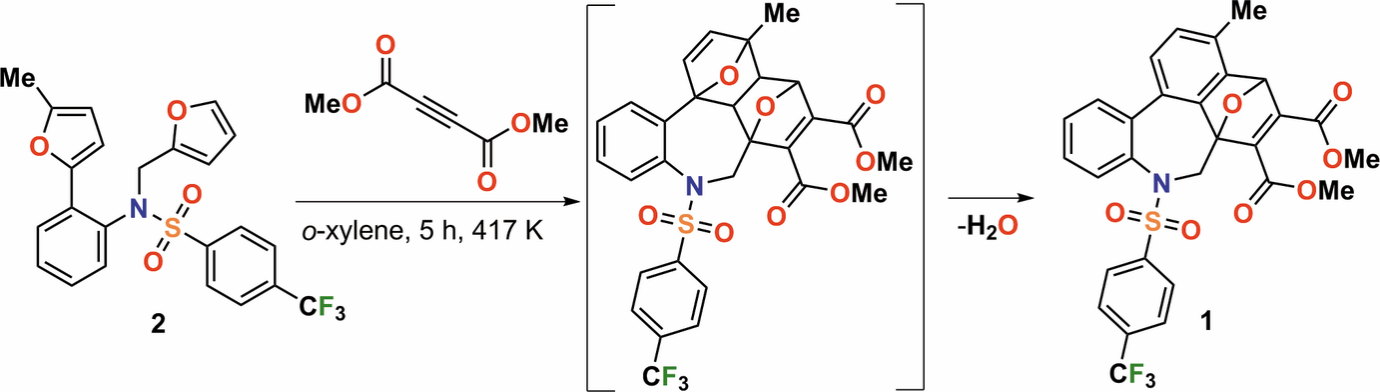
Synthesis of dimethyl 3-methyl-8-{[4-(tri­fluoro­meth­yl)phen­yl]sulfon­yl}-7,8-di­hydro-4*H*-4,6a-ep­oxy­benzo[*b*]naphtho­[1,8-*de*]azepine-5,6-di­carboxyl­ate.

7-Oxabi­cyclo­[2.2.1]heptenes, products of the thermic reaction between furans and alkenes or alkynes, have great synthetic potential as a useful tool for the design of a broad diversity of substances with various practical properties. For example, these scaffolds can be used in the synthesis of polycyclic arenes – fragments of graphene – and serve as models for new carbon-based electronic materials (Eda *et al.*, 2015 ▸; Criado *et al.*, 2013 ▸; Furrer *et al.*, 2013 ▸). The 7-oxabi­cyclo­[2.2.1]heptane moiety annelated with other rings serves as a scaffold for the preparation of mol­ecular tweezers (Murphy *et al.*, 2016 ▸; Warrener *et al.*, 1999 ▸), supra­molecular systems (Chou *et al.*, 2015 ▸; Oh *et al.*, 2010 ▸; Eckert-Maksić *et al.*, 2005 ▸), bridging donor–acceptor mol­ecules (Chakrabarti *et al.*, 2007 ▸), various bioactive and natural products (Roscalesa *et al.*, 2017 ▸; Enev *et al.*, 2012 ▸; Gromov *et al.*, 2009 ▸; Schindler *et al.*, 2009 ▸; Vogel *et al.*, 1999 ▸), high-mol­ecular-weight materials (Margetić *et al.*, 2010 ▸; Warrener *et al.*, 2001 ▸; Vogel *et al.*, 1999 ▸), *etc*. Under acid catalysis and temperature, cyclo­addition inter­mediates can be converted into phenols, cyclo­hexenoles, or substituted aromatic hydro­carbons (Zaytsev *et al.*, 2019 ▸; Zubkov *et al.*, 2012*a* ▸,*b* ▸; Guliyeva *et al.*, 2024 ▸). Continuing our research into the chemistry of furyl-substituted sulfonamides (Burkin *et al.*, 2024 ▸; Mammadova *et al.*, 2023*a* ▸,*b* ▸), a new approach toward the cyclo­addition of dimethyl but-2-ynedioate (DMAD) with substituted furans (Zubkov *et al.*, 2009 ▸; Borisova *et al.*, 2018*a* ▸,*b* ▸) has been developed. In particular, in the course of the thermic [4 + 2] cyclo­addition between DMAD and sulfamide **2**, an inter­esting sequence of reaction steps was observed; [4 + 2] cyclo­addition, cleavage of the ep­oxy bridge, and a subsequent aromatization of the cyclo­hexene ring (Fig. 1 ▸).
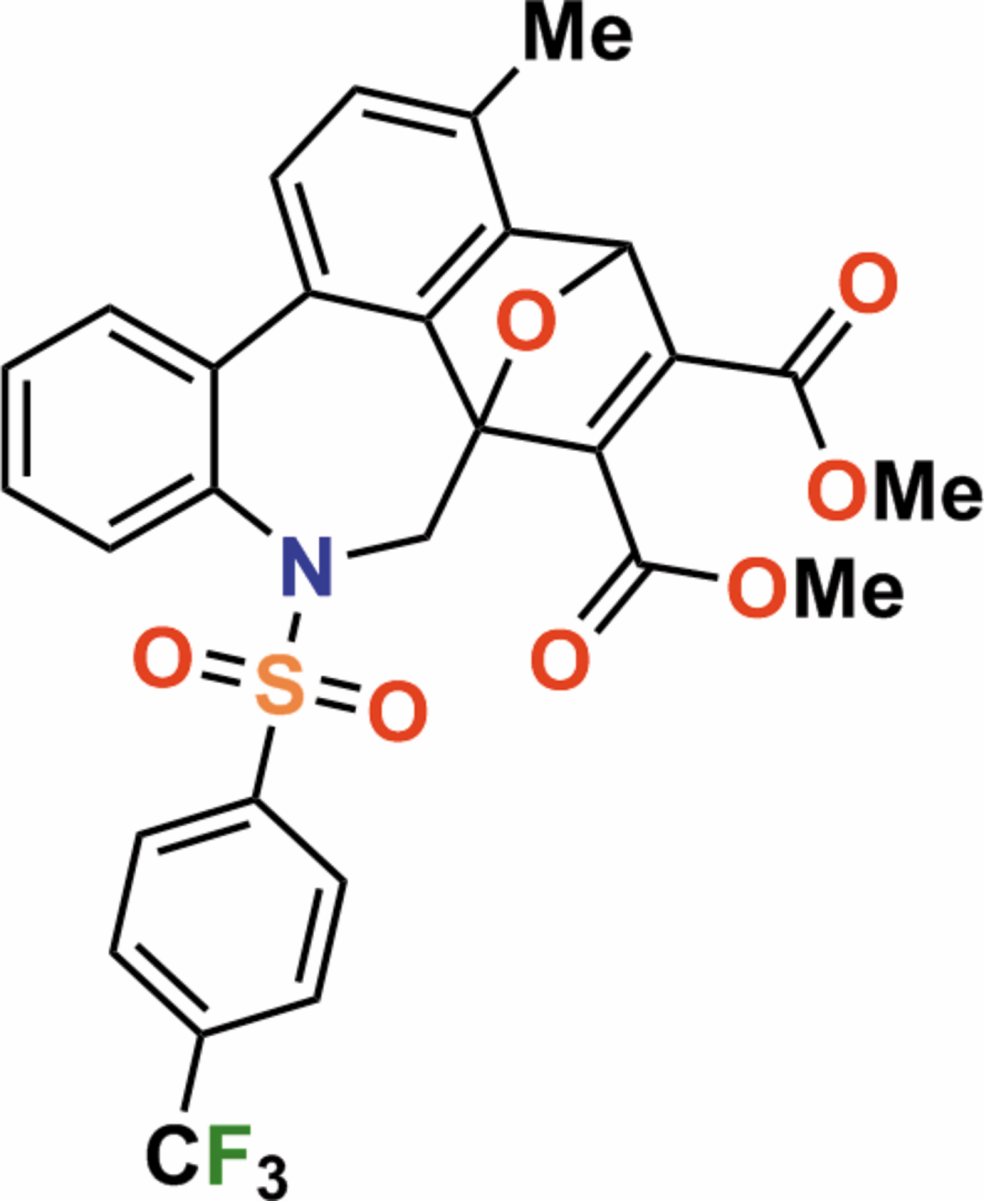


## 2. Structural commentary

**Figure 2:**
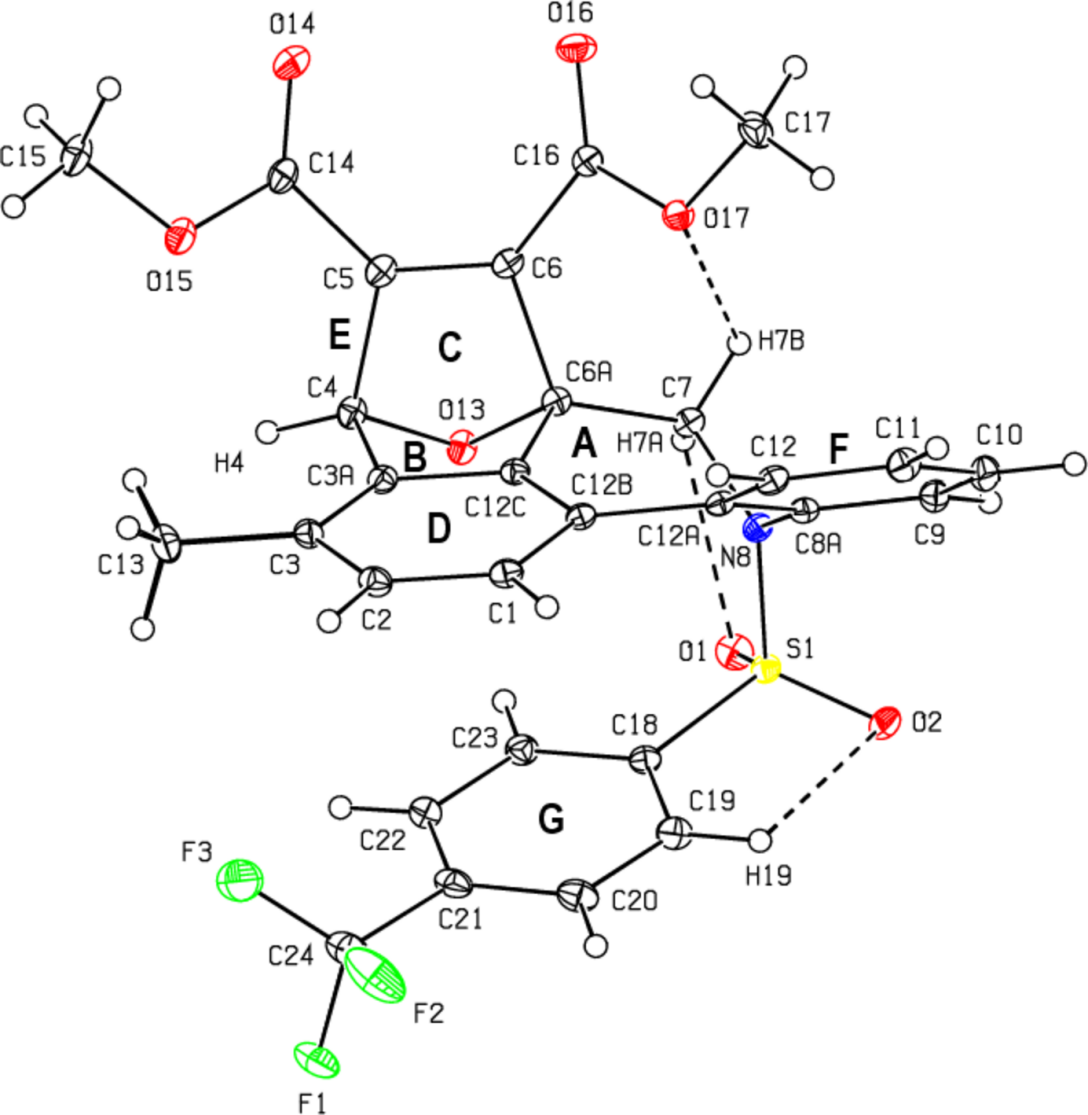
Mol­ecular structure of the title compound showing atom labelling and ellipsoids at the 30% probability level. The minor disorder component has been omitted for clarity.

**Figure 3:**
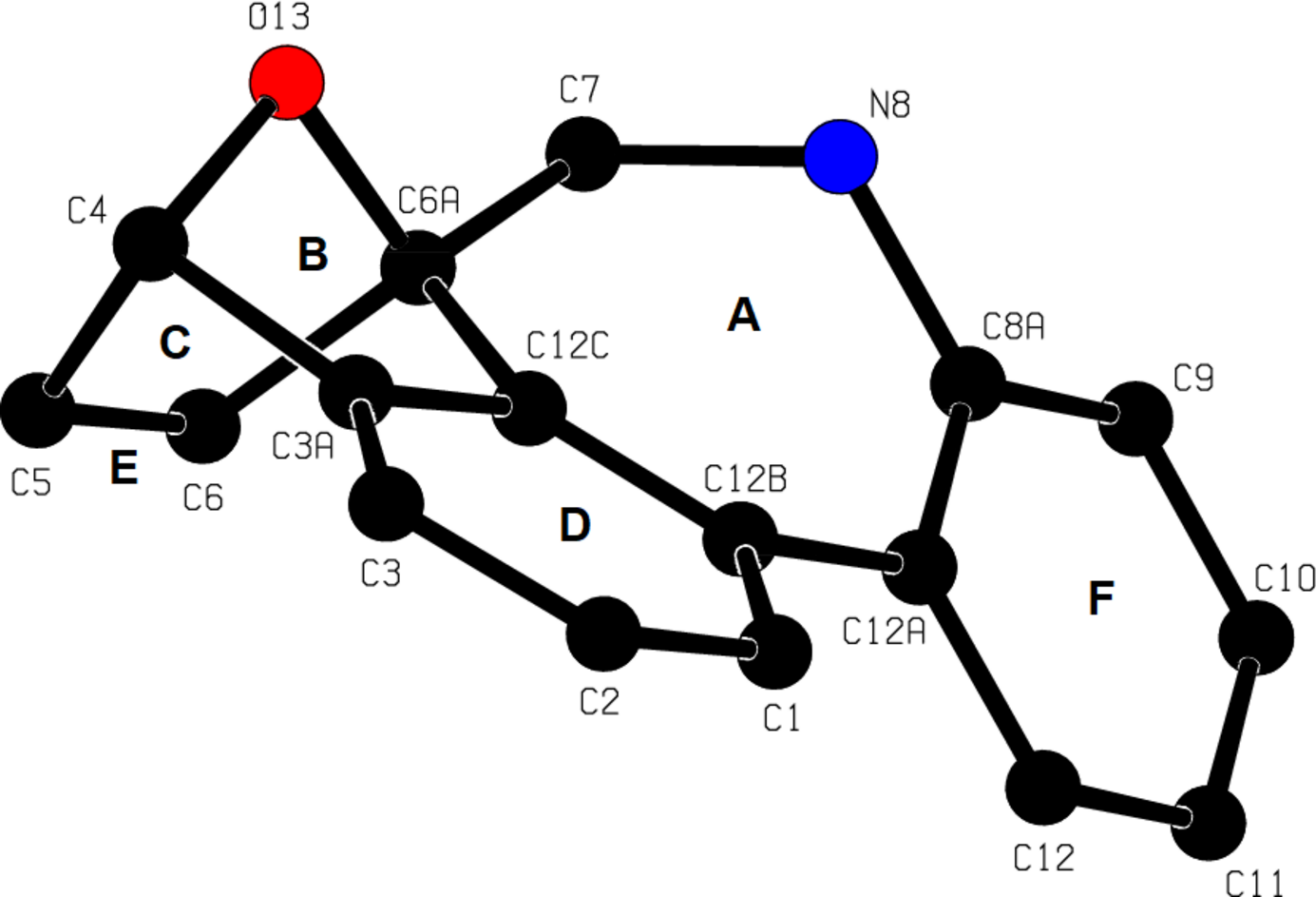
A detailed view of the central rings of the title mol­ecule.

Fig. 2 ▸ shows the mol­ecular structure of the title compound, intra­molecular C—H⋯O hydrogen bonds, and naming of the rings in the mol­ecule. The mol­ecular conformation is stable due to the intra­molecular hydrogen bonds C7—H7*B*⋯O17, C7—H7*A*⋯O1 and C19—H19⋯O2, which form *S*(6), *S*(5) and *S*(5) ring motifs, respectively (Fig. 2 ▸; Table 1 ▸; Bernstein *et al.*, 1995 ▸). Fig. 3 ▸ shows a detailed view of the central rings of the mol­ecule. The central ring *A* (C6*A*/C7/N8/C8*A*/C12*A*/C12*B*/C12*C*) exhibits a distorted chair form [puckering parameters: *q*2 = 0.708 (1), *q*3 = 207 (1) Å, φ(2) = −29.76 (9), φ(3) = −138.1 (4) °, *Q*_T_ = 0.738 (1) Å, and spherical polar angle θ(2) = 73.70 (9)°]. Ring *A* (r.m.s. deviation of fitted atoms = 0.2783 Å) subtends dihedral angles of 20.58 (5), 50.46 (5), 30.64 (5) and 28.18 (5)°, respectively, with rings *D* (C1–C3/C3*A*/C12*C*/C12*B*), *E* (C3*A*/C4–C6/C6*A*/C12*C*), *F* (C8*A*/C9–C12/C12*A*) and *G* (C18–C23). In the 7-oxabi­cyclo­[2.2.1]hepta-2,5-diene unit, the five-membered rings *B* (O13/C4/C3*A*/C12*C*/C6*A*) and *C* (O13/C4–C6/C6*A*) show envelope conformations on atom O13 [*B*: *q*(2) = 0.5436 (12) Å, φ(2) = 0.35 (14)° and *C*: *q*(2) = 0.5395 (12) Å, φ(2) = 179.95 (14)°]. The bond lengths and angles in the title compound are in good agreement with those reported for related compounds (see *Database survey* section).

## 3. Supra­molecular features and Hirshfeld surface analysis

**Figure 4:**
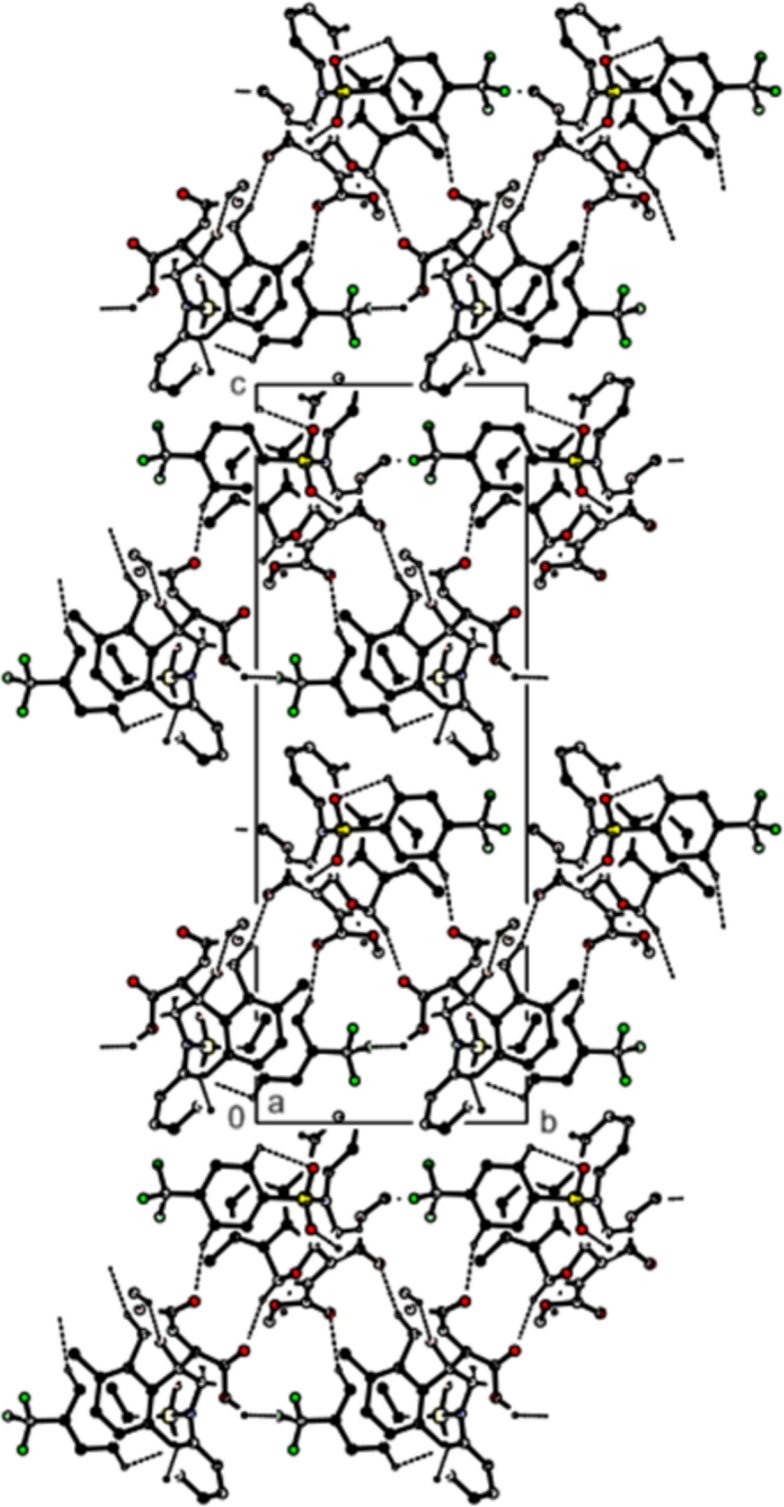
A view along the *a* axis of the title compound, showing the crystal packing. C—H⋯O and C—H⋯F hydrogen bonds are shown as dashed lines; H atoms not involved in hydrogen bonding have been omitted.

**Figure 5:**
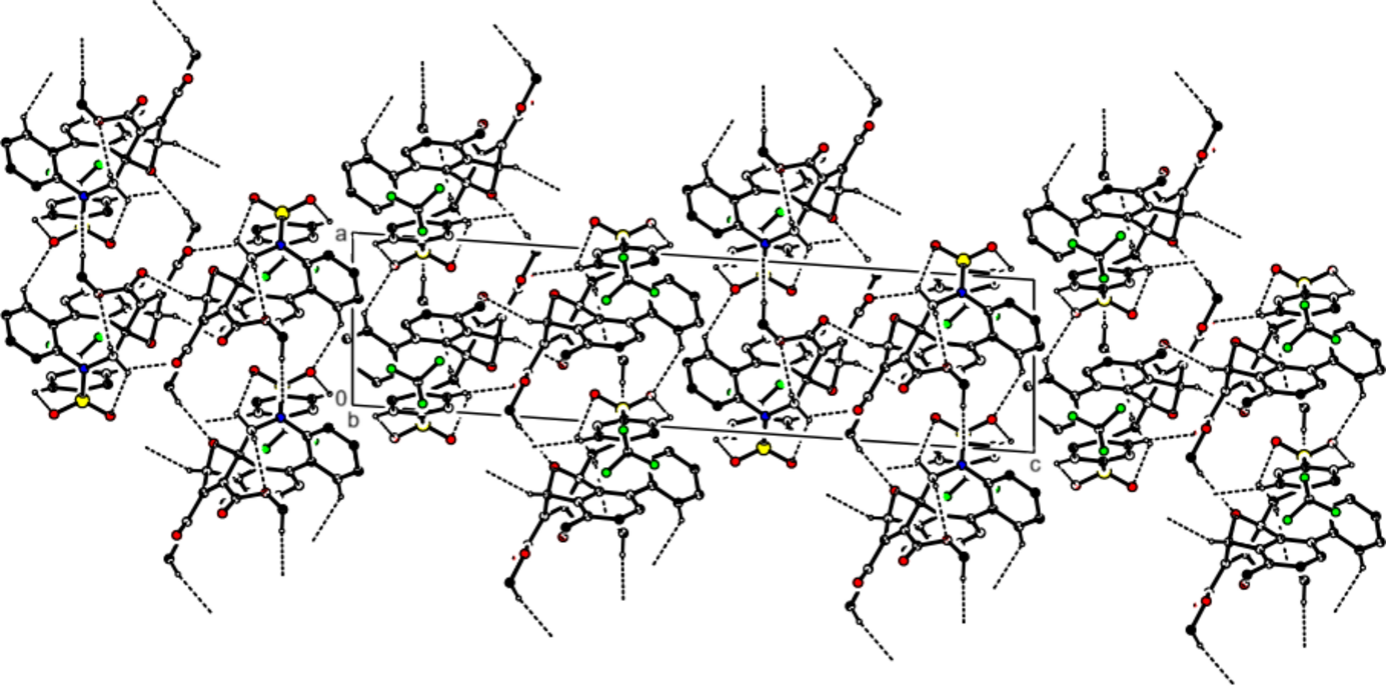
A view along the *b* axis of the title compound, showing the crystal packing. C—H⋯O and C—H⋯F hydrogen bonds are shown as dashed lines; H atoms not involved in hydrogen bonding have been omitted.

**Figure 6:**
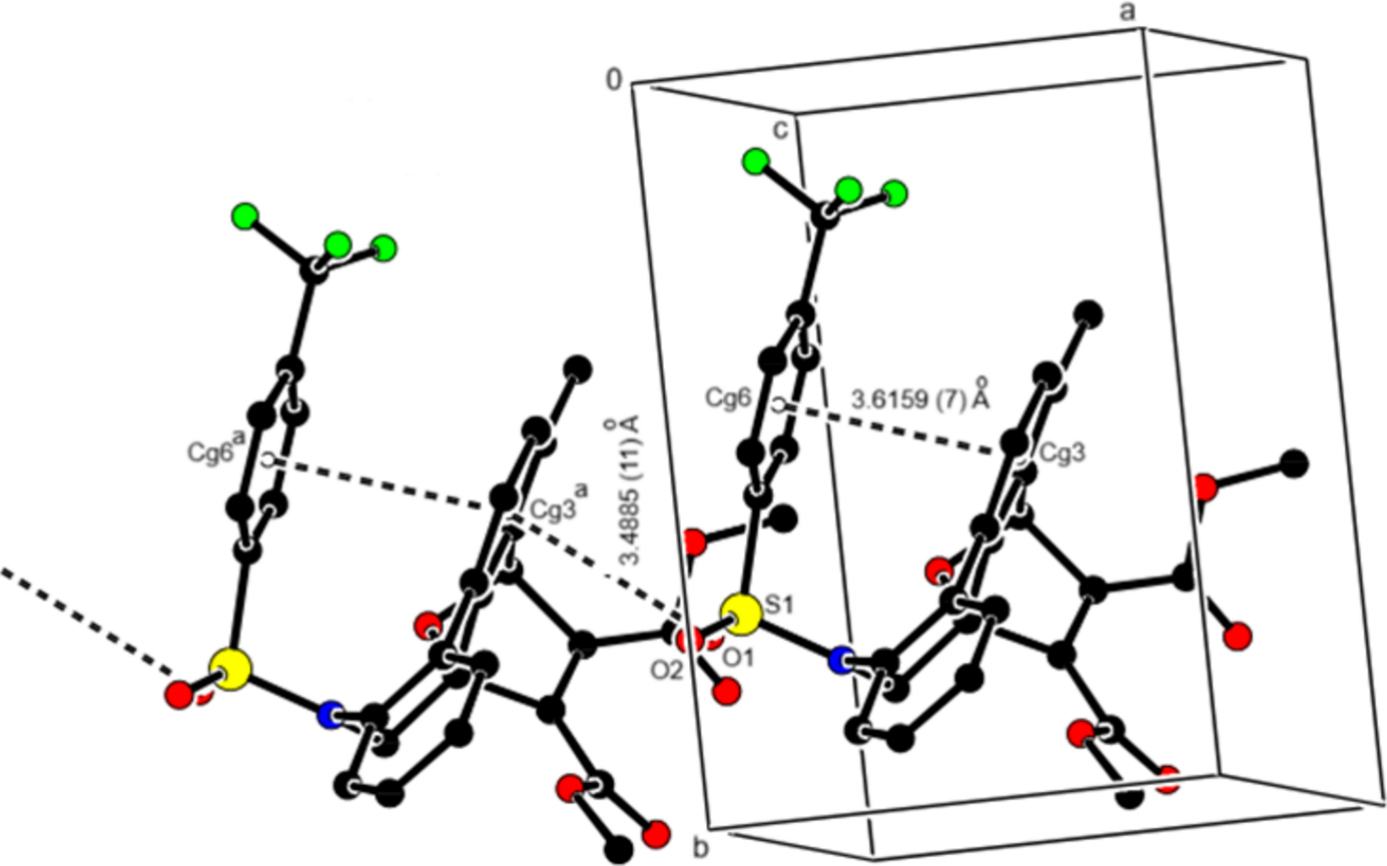
A partial packing diagram showing S—O⋯π and π–π inter­actions as dashed lines. H atoms not involved in hydrogen bonding have been omitted.

In the crystal, mol­ecules form 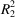
(17) ring motifs by C—H⋯O inter­actions and are linked by C—H⋯F inter­actions to form sheets parallel to the (002) plane (Figs. 4 ▸ and 5 ▸; Table 1 ▸). Additionally, S—O⋯π (Fig. 5 ▸; Table 1 ▸) and π–π inter­actions [Fig. 6 ▸; *Cg*3⋯*Cg*6 = 3.6159 (7) Å, slippage = 0.804 Å; where *Cg*3 and *Cg*6 are the centroids of rings *D* (C1–C3/C3*A*/C12*C*/C12*B*) and *G* (C18–C23), respectively] link the mol­ecules along the *a-*axis direction. van der Waals inter­actions between the mol­ecular sheets reinforce the mol­ecular packing.

**Figure 7:**
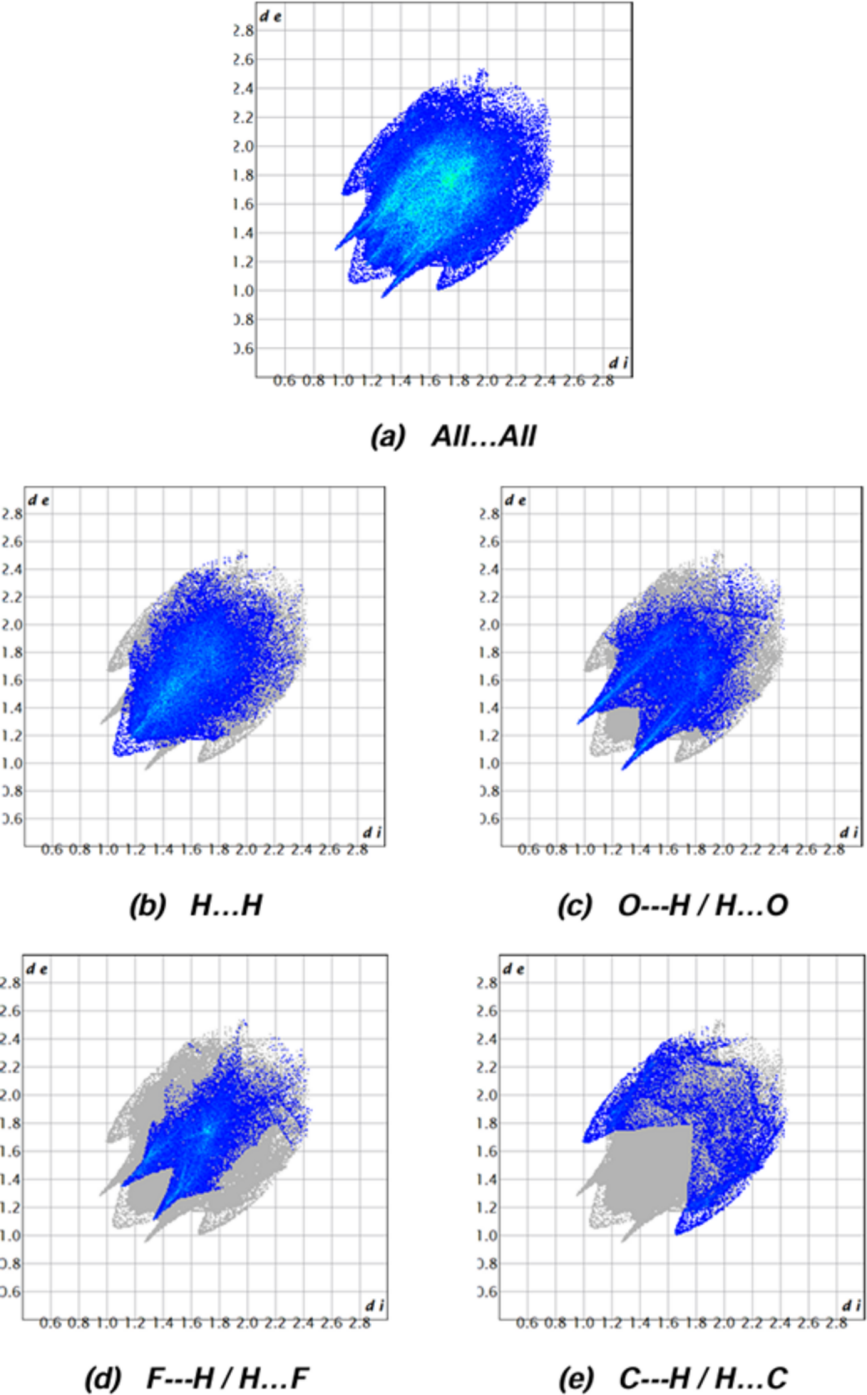
(*a*) The full two-dimensional fingerprint plot for the title compound and those delineated into (*b*) H⋯H, (*c*) O⋯H/H⋯O, (*c*) F⋯H/H⋯F and (*c*) C⋯H/H⋯C contacts.

Hirshfeld surfaces and the corresponding two-dimensional fingerprint plots were created using *CrystalExplorer17.5* (Spackman *et al.*, 2021 ▸) in order to visualize the inter­molecular inter­actions (Tables 1 ▸ and 2 ▸). Fig. 7 ▸ shows the full two-dimensional fingerprint plot and those delineated into the major contacts: H⋯H (37.3%), O⋯H/H⋯O (24.1%), F⋯H/H⋯F (19.0%) and C⋯H/H⋯C (10.3%). Smaller contributions are made by O⋯C/C⋯O(4.9%), O⋯O(1.6%), C⋯C (1.5%), F⋯C/C⋯F (0.7%), F⋯O/O⋯F (0.4%), N⋯H/H⋯N (0.2%), S⋯C/C⋯S (0.1%) and S⋯H/H⋯S (0.1%) inter­actions.

## 4. Database survey

A search of the Cambridge Structural Database (Version 5.41, last update November 2019; Groom *et al.*, 2016 ▸) for 11-oxatri­cyclo­[6.2.1.0^2,7^]undeca­nes gave 739 hits, while a search for 3-methyl-11-oxatri­cyclo­[6.2.1.0^2,7^]undeca­nes gave zero hits. In these searches, the most related compounds are CSD refcode COKHAP (Sadikhova *et al.*, 2024 ▸) and POYBEL (Zubkov *et al.*, 2009 ▸). In COKHAP, two hexane rings and one oxane ring are fused together. The two hexane rings tend toward a distorted boat conformation, while the tetra­hydro­furan and di­hydro­furan rings adopt envelope conformations. The oxane ring is puckered. In the crystal, C—H⋯O hydrogen bonds connect the mol­ecules into a three-dimensional network. POYBEL comprises a fused penta­cyclic system containing two five-membered (cyclo­pentane and tetra­hydro­furan) and three six-membered (tetra­hydro­pyridinone, tetra­hydro­pyridine and benzene) rings. Both five-membered rings of the bicyclic fragment have the usual envelope conformations, and the two central six-membered rings adopt sofa and non-symmetrical half-chair conformations.

In addition, three related compounds containing the O=S=O group are YIKROD (Mammadova *et al.*, 2023*a* ▸), KETGID (Schinke *et al.*, 2022 ▸) and LUJKUA (Yakuth *et al.*, 2024 ▸). In YIKROD, intra­molecular inter­actions are observed between the furan and benzene rings of the 4-cyano­phenyl group. In the crystal, mol­ecules are connected *via* C—H⋯O and C—H⋯N hydrogen bonds, forming layers parallel to the (100) plane. These layers are inter­connected by C⋯H inter­actions and weak van der Waals inter­actions. In KETGID, the 1,2-oxazole and methanone fragments form an almost coplanar unit. The crystal structure features three short inter­molecular C—H⋯O contacts involving the methane­sulfonyl-O atoms. In LUJKUA, the asymmetric unit contains two distinct mol­ecules, which exhibit differences in conformation resulting from a variation in key torsion angles. These distinctions influence the mol­ecular orientation and inter­molecular inter­actions, with strong N—H⋯N and N—H⋯O hydrogen bonds forming a centrosymmetric tetra­mer stabilized by π–π stacking.

## 5. Synthesis and crystallization

Dimethyl but-2-ynedioate (133.2 µL, 1.1 mmol) was added to a solution of *N*-(furan-2-ylmeth­yl)-*N*-[2-(5-methyl­furan-2-yl)phen­yl]-4-(tri­fluoro­meth­yl)benzene­sulfonamide **2** (100 mg, 0.22 mmol) in *o*-xylene (5 mL). The mixture was refluxed for 5 h. After cooling of the reaction to r.t, the solvent was evaporated under reduced pressure and the crude product was purified by column chromatography (eluent: from hexane to ethyl acetate). The title compound was obtained as colourless powder, yield 27%, 35 mg (0.059 mmol); m.p. 486–487 K. A single crystal of the title compound was grown from ethanol. IR (KBr), *ν* (cm^−1^): 1753 (CO_2_), 1325 (ν_as_ SO_2_), 1169 (ν_s_ SO_2_). ^1^H NMR (700.2 MHz, CDCl_3_) (*J*, Hz): *δ* 7.71 (*dd*, *J* = 7.6, 1.7, 1H, H Ar), 7.50–7.44 (*m*, 5H, H Ar), 7.20 (*d*, *J* = 8.1, 2H, H Ar), 6.69 (*d*, *J* = 7.9, 1H, H Ar), 6.61 (*d*, *J* = 8.1, 1H, H Ar), 5.91 (*s*, 1H, H Ar), 5.15 (*d*, *J* = 16.7, 1H, NC*H*), 4.47 (*d*, *J* = 16.7, 1H, NC*H*), 3.76 (*s*, 3H, OCH_3_), 3.47 (*s*, 3H, OCH_3_), 2.29 (*s*, 3H, CH_3_). ^13^C{^1^H} NMR (176.1 MHz, CDCl_3_): *δ* there are no signal of CF_3_ 163.1, 162.4, 151.2, 150.6, 145.3, 144.1, 142.7, 137.4, 137.0, 133.3 (*q*, *J* = 32.4, 1 C), 132.4, 130.9, 130.0, 129.7, 129.2 (2C), 128.3, 127.9 (2C), 126.1, 124.5 (*q*, *J* = 4.1, 2 C), 96.9, 81.3, 54.8, 52.5, 52.2, 17.4. ^19^F{^1^H} NMR (658.8 MHz, CDCl_3_): −63.27. MS (ESI) *m/z*: [*M* + H]^+^ 586. Elemental analysis calculated (%) for C_29_H_22_F_3_NO_7_S: C 59.49, H 3.79, N 2.39, S 5.48; found: C 59.81, H 3.48, N 2.19, S 5.33.

## 6. Refinement

Crystal data, data collection and structure refinement details are summarized in Table 3 ▸. All C-bound H atoms were positioned geometrically (C—H = 0.95 and 1.00 Å) and refined using a riding model with *U*_iso_(H) = 1.2 or 1.5*U*_eq_(C). The methyl group (C13) attached to the benzene ring was found to be disordered over two positions with a refined occupancy ratio of 0.53 (2): 0.47 (2). A SADI instruction was used to restrain the C3—C13 and C3—C13′ bonds. The anisotropic displacement parameters of both parts of the carbon atom of the disordered methyl group were restrained to be similar with EADP instruction.

## Supplementary Material

Crystal structure: contains datablock(s) I. DOI: 10.1107/S2056989025004426/nx2026sup1.cif

Structure factors: contains datablock(s) I. DOI: 10.1107/S2056989025004426/nx2026Isup2.hkl

CCDC reference: 2451675

Additional supporting information:  crystallographic information; 3D view; checkCIF report

## Figures and Tables

**Table 1:** Hydrogen-bond geometry (Å, °)

*D*—H⋯*A*	*D*—H	H⋯*A*	*D*⋯*A*	*D*—H⋯*A*
C4—H4⋯O16^i^	1.00	2.49	3.3893 (16)	149
C7—H7*A*⋯O1	0.99	2.35	2.8481 (15)	111
C7—H7*B*⋯O17	0.99	2.46	3.0071 (14)	115
C12—H12⋯O2^ii^	0.95	2.55	3.1357 (15)	120
C15—H15*A*⋯O13^ii^	0.98	2.47	3.3741 (17)	153
C17—H17*B*⋯F1^iii^	0.98	2.54	3.3033 (17)	135
C19—H19⋯O2	0.95	2.52	2.9003 (17)	104
C22—H22⋯O14^i^	0.95	2.37	3.2698 (19)	158

Symmetry codes: (i) 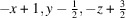; (ii) 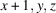; (iii) 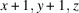.

**Table 2:** Summary of short inter­atomic contacts (Å) in the title compound

Contact	distance	Symmetry operation
F3⋯H17*C*	2.72	*x*, −1 + *y*, *z*
F1⋯H17*B*	2.54	−1 + *x*, −1 + *y*, *z*
H12⋯H1	2.54	1 − *x*, 1 − *y*, 1 − *z*
O13⋯H15*A*	2.47	−1 + *x*, *y*, *z*
O14⋯H22	2.37	1 − *x*, 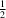 + *y*, 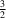 − *z*
O14⋯H15*B*	2.64	2 − *x*, 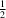 + *y*, 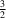 − *z*
H22⋯O14	2.37	1 − *x*, − 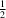 + *y*, 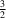 − *z*
H19⋯H19	2.27	−*x*, 1 − *y*, 1 − *z*
H17*A*⋯H10	2.51	1 − *x*, 2 − *y*, 1 − *z*
H10⋯H9	2.58	1 − *x*, 2 − *y*, 1 − *z*

**Table 3:** Experimental details

Crystal data	
Chemical formula	C_29_H_22_F_3_NO_7_S
*M*_r_	585.53
Crystal system, space group	Monoclinic, *P*2_1_/*c*
Temperature (K)	100
*a*, *b*, *c* (Å)	7.6375 (5), 11.0324 (6), 30.2019 (8)
β (°)	93.983 (1)
*V* (Å^3^)	2538.7 (2)
*Z*	4
Radiation type	Cu *K*α
μ (mm^−1^)	1.79
Crystal size (mm)	0.35 × 0.18 × 0.17

Data collection	
Diffractometer	Rigaku XtaLAB Synergy-S, HyPix-6000HE area-detector
Absorption correction	Multi-scan (*CrysAlis PRO*; Rigaku OD, 2021 ▸)
*T*_min_, *T*_max_	0.713, 0.737
No. of measured, independent and observed [*I* > 2σ(*I*)] reflections	31570, 5526, 5187
*R*_int_	0.049
(sin θ/λ)_max_ (Å^−1^)	0.639

Refinement	
*R*[*F*^2^ > 2σ(*F*^2^)], *wR*(*F*^2^), *S*	0.037, 0.103, 1.05
No. of reflections	5526
No. of parameters	379
No. of restraints	1
H-atom treatment	H-atom parameters constrained
Δρ_max_, Δρ_min_ (e Å^−3^)	0.45, −0.43

Computer programs: *CrysAlis PRO* (Rigaku OD, 2021 ▸), *SHELXT2016/6* (Sheldrick, 2015*a* ▸), *SHELXL2016/6* (Sheldrick, 2015*b* ▸, *ORTEP-3 for Windows* (Farrugia, 2012 ▸) and *PLATON* (Spek, 2020 ▸).

## supplementary crystallographic information

### Dimethyl 3-methyl-8-{[4-(trifluoromethyl)phenyl]sulfonyl}-7,8-dihydro-4H-4,6a-epoxybenzo[b]naphtho[1,8-de]azepine-5,6-dicarboxylate Crystal data

**Table:**

C_29_H_22_F_3_NO_7_S	*F*(000) = 1208
*M**_r_* = 585.53	*D*_x_ = 1.532 Mg m^−^^3^
Monoclinic, *P*2_1_/*c*	Cu *K*α radiation, λ = 1.54184 Å
*a* = 7.6375 (5) Å	Cell parameters from 20964 reflections
*b* = 11.0324 (6) Å	θ = 2.9–79.9°
*c* = 30.2019 (8) Å	µ = 1.79 mm^−^^1^
β = 93.983 (1)°	*T* = 100 K
*V* = 2538.7 (2) Å^3^	Prism, colourless
*Z* = 4	0.35 × 0.18 × 0.17 mm

### Dimethyl 3-methyl-8-{[4-(trifluoromethyl)phenyl]sulfonyl}-7,8-dihydro-4H-4,6a-epoxybenzo[b]naphtho[1,8-de]azepine-5,6-dicarboxylate Data collection

**Table:**

Rigaku XtaLAB Synergy-S, HyPix-6000HE area-detector diffractometer	5187 reflections with *I* > 2σ(*I*)
Radiation source: micro-focus sealed X-ray tube	*R*_int_ = 0.049
φ and ω scans	θ_max_ = 80.1°, θ_min_ = 2.9°
Absorption correction: multi-scan (CrysAlisPro; Rigaku OD, 2021)	*h* = −9→8
*T*_min_ = 0.713, *T*_max_ = 0.737	*k* = −14→13
31570 measured reflections	*l* = −38→38
5526 independent reflections	

### Dimethyl 3-methyl-8-{[4-(trifluoromethyl)phenyl]sulfonyl}-7,8-dihydro-4H-4,6a-epoxybenzo[b]naphtho[1,8-de]azepine-5,6-dicarboxylate Refinement

**Table:**

Refinement on *F*^2^	Secondary atom site location: difference Fourier map
Least-squares matrix: full	Hydrogen site location: inferred from neighbouring sites
*R*[*F*^2^ > 2σ(*F*^2^)] = 0.037	H-atom parameters constrained
*wR*(*F*^2^) = 0.103	*w* = 1/[σ^2^(*F*_o_^2^) + (0.0633*P*)^2^ + 0.8141*P*] where *P* = (*F*_o_^2^ + 2*F*_c_^2^)/3
*S* = 1.05	(Δ/σ)_max_ = 0.001
5526 reflections	Δρ_max_ = 0.45 e Å^−^^3^
379 parameters	Δρ_min_ = −0.43 e Å^−^^3^
1 restraint	Extinction correction: SHELXL-2019/2 (Sheldrick, 2015b), Fc^*^=kFc[1+0.001xFc^2^λ^3^/sin(2θ)]^-1/4^
Primary atom site location: difference Fourier map	Extinction coefficient: 0.00089 (14)

### Dimethyl 3-methyl-8-{[4-(trifluoromethyl)phenyl]sulfonyl}-7,8-dihydro-4H-4,6a-epoxybenzo[b]naphtho[1,8-de]azepine-5,6-dicarboxylate Special details

**Table:**

Geometry. All esds (except the esd in the dihedral angle between two l.s. planes) are estimated using the full covariance matrix. The cell esds are taken into account individually in the estimation of esds in distances, angles and torsion angles; correlations between esds in cell parameters are only used when they are defined by crystal symmetry. An approximate (isotropic) treatment of cell esds is used for estimating esds involving l.s. planes.

### Dimethyl 3-methyl-8-{[4-(trifluoromethyl)phenyl]sulfonyl}-7,8-dihydro-4H-4,6a-epoxybenzo[b]naphtho[1,8-de]azepine-5,6-dicarboxylate Fractional atomic coordinates and isotropic or equivalent isotropic displacement parameters (Å^2^)

**Table:**

	*x*	*y*	*z*	*U*_iso_*/*U*_eq_	Occ. (<1)
S1	−0.08268 (3)	0.68046 (3)	0.60331 (2)	0.01536 (10)	
F1	0.03672 (12)	0.08285 (8)	0.60261 (3)	0.0324 (2)	
F2	0.22478 (16)	0.13678 (9)	0.55695 (5)	0.0501 (3)	
F3	0.29014 (14)	0.14333 (10)	0.62756 (5)	0.0539 (3)	
O1	−0.16062 (12)	0.70213 (9)	0.64434 (3)	0.0216 (2)	
O2	−0.17402 (12)	0.70996 (9)	0.56162 (3)	0.0225 (2)	
C1	0.48862 (15)	0.49339 (11)	0.57852 (4)	0.0171 (2)	
H1	0.496038	0.479440	0.547665	0.021*	
C2	0.55556 (16)	0.40746 (11)	0.60859 (4)	0.0187 (2)	
H2	0.607100	0.336071	0.597644	0.022*	
C3	0.54990 (15)	0.42217 (11)	0.65483 (4)	0.0172 (2)	
C3A	0.47063 (15)	0.52723 (11)	0.66816 (4)	0.0156 (2)	
C4	0.44151 (15)	0.58212 (12)	0.71373 (4)	0.0171 (2)	
H4	0.444763	0.524274	0.739264	0.021*	
C5	0.56803 (16)	0.69088 (11)	0.71852 (4)	0.0165 (2)	
C6	0.50225 (15)	0.77414 (11)	0.68956 (4)	0.0149 (2)	
C6A	0.33411 (15)	0.71572 (11)	0.66698 (4)	0.0141 (2)	
C7	0.18668 (15)	0.79745 (11)	0.64834 (4)	0.0158 (2)	
H7A	0.096592	0.803245	0.670287	0.019*	
H7B	0.234316	0.879823	0.644221	0.019*	
N8	0.10246 (13)	0.75554 (9)	0.60592 (3)	0.0148 (2)	
C8A	0.19749 (15)	0.76703 (11)	0.56657 (4)	0.0144 (2)	
C9	0.14221 (16)	0.85518 (12)	0.53567 (4)	0.0186 (2)	
H9	0.041411	0.902688	0.540357	0.022*	
C10	0.23335 (17)	0.87407 (12)	0.49810 (4)	0.0217 (3)	
H10	0.195141	0.934034	0.477015	0.026*	
C11	0.38118 (17)	0.80441 (12)	0.49158 (4)	0.0202 (3)	
H11	0.445235	0.817581	0.466155	0.024*	
C12	0.43538 (16)	0.71573 (12)	0.52212 (4)	0.0171 (2)	
H12	0.535760	0.668175	0.517074	0.020*	
C12A	0.34499 (15)	0.69485 (11)	0.56029 (4)	0.0143 (2)	
C12B	0.40963 (14)	0.60123 (11)	0.59260 (4)	0.0144 (2)	
C12C	0.40171 (14)	0.61400 (11)	0.63793 (4)	0.0137 (2)	
O13	0.27786 (11)	0.64524 (8)	0.70381 (3)	0.01706 (19)	
C13	0.6235 (19)	0.3275 (12)	0.6870 (4)	0.0238 (3)	0.53 (2)
H13A	0.732679	0.294903	0.676576	0.036*	0.53 (2)
H13B	0.538176	0.261703	0.689067	0.036*	0.53 (2)
H13C	0.647483	0.364271	0.716356	0.036*	0.53 (2)
C13'	0.625 (2)	0.3275 (13)	0.6867 (5)	0.0238 (3)	0.47 (2)
H13D	0.747394	0.348027	0.695799	0.036*	0.47 (2)
H13E	0.621140	0.248114	0.672104	0.036*	0.47 (2)
H13F	0.556513	0.324858	0.712874	0.036*	0.47 (2)
C14	0.74252 (17)	0.68539 (12)	0.74318 (4)	0.0187 (3)	
O14	0.82551 (14)	0.77048 (10)	0.75805 (4)	0.0298 (2)	
O15	0.79316 (12)	0.56938 (9)	0.74709 (3)	0.0221 (2)	
C15	0.96586 (17)	0.54877 (14)	0.76935 (5)	0.0252 (3)	
H15A	1.051895	0.601521	0.756353	0.038*	
H15B	0.999568	0.463854	0.765660	0.038*	
H15C	0.962354	0.567077	0.801032	0.038*	
C16	0.58807 (15)	0.88141 (11)	0.67119 (4)	0.0160 (2)	
O16	0.68066 (13)	0.95432 (9)	0.69113 (3)	0.0247 (2)	
O17	0.54422 (11)	0.88538 (8)	0.62730 (3)	0.01773 (19)	
C17	0.63324 (19)	0.97498 (13)	0.60257 (5)	0.0244 (3)	
H17A	0.585829	0.974193	0.571579	0.037*	
H17B	0.759006	0.956514	0.603902	0.037*	
H17C	0.615553	1.055289	0.615390	0.037*	
C18	−0.02522 (15)	0.52580 (11)	0.60113 (4)	0.0161 (2)	
C19	−0.01841 (17)	0.47033 (12)	0.56005 (4)	0.0213 (3)	
H19	−0.055255	0.512804	0.533666	0.026*	
C20	0.04285 (18)	0.35202 (13)	0.55788 (5)	0.0241 (3)	
H20	0.049166	0.312688	0.530058	0.029*	
C21	0.09484 (17)	0.29210 (12)	0.59721 (5)	0.0220 (3)	
C22	0.08252 (17)	0.34703 (12)	0.63835 (5)	0.0224 (3)	
H22	0.115329	0.303757	0.664827	0.027*	
C23	0.02216 (17)	0.46516 (12)	0.64049 (4)	0.0195 (3)	
H23	0.013327	0.503979	0.668321	0.023*	
C24	0.16202 (19)	0.16488 (13)	0.59579 (6)	0.0296 (3)	

### Dimethyl 3-methyl-8-{[4-(trifluoromethyl)phenyl]sulfonyl}-7,8-dihydro-4H-4,6a-epoxybenzo[b]naphtho[1,8-de]azepine-5,6-dicarboxylate Atomic displacement parameters (Å^2^)

**Table:**

	*U*^11^	*U*^22^	*U*^33^	*U*^12^	*U*^13^	*U*^23^
S1	0.01088 (15)	0.01765 (16)	0.01767 (16)	0.00027 (9)	0.00183 (11)	0.00049 (10)
F1	0.0335 (5)	0.0180 (4)	0.0464 (5)	−0.0065 (3)	0.0085 (4)	−0.0001 (4)
F2	0.0591 (7)	0.0245 (5)	0.0717 (8)	0.0035 (4)	0.0407 (6)	−0.0050 (5)
F3	0.0374 (5)	0.0263 (5)	0.0941 (10)	0.0095 (4)	−0.0236 (6)	−0.0078 (5)
O1	0.0165 (4)	0.0240 (5)	0.0252 (5)	0.0012 (3)	0.0086 (4)	−0.0018 (4)
O2	0.0159 (4)	0.0257 (5)	0.0250 (5)	−0.0014 (3)	−0.0049 (3)	0.0034 (4)
C1	0.0160 (5)	0.0185 (6)	0.0170 (5)	−0.0007 (4)	0.0020 (4)	−0.0035 (4)
C2	0.0174 (5)	0.0157 (5)	0.0231 (6)	0.0007 (4)	0.0027 (4)	−0.0026 (4)
C3	0.0137 (5)	0.0166 (6)	0.0213 (6)	−0.0015 (4)	0.0013 (4)	0.0031 (4)
C3A	0.0129 (5)	0.0186 (6)	0.0154 (5)	−0.0025 (4)	0.0017 (4)	0.0016 (4)
C4	0.0153 (5)	0.0215 (6)	0.0148 (5)	0.0005 (4)	0.0025 (4)	0.0028 (4)
C5	0.0169 (6)	0.0218 (6)	0.0110 (5)	0.0010 (4)	0.0028 (4)	−0.0018 (4)
C6	0.0143 (5)	0.0193 (6)	0.0114 (5)	0.0005 (4)	0.0023 (4)	−0.0036 (4)
C6A	0.0137 (5)	0.0174 (5)	0.0116 (5)	−0.0014 (4)	0.0035 (4)	0.0002 (4)
C7	0.0142 (5)	0.0178 (5)	0.0155 (5)	0.0011 (4)	0.0013 (4)	−0.0029 (4)
N8	0.0123 (4)	0.0175 (5)	0.0144 (5)	−0.0002 (4)	0.0012 (4)	−0.0006 (4)
C8A	0.0133 (5)	0.0157 (5)	0.0141 (5)	−0.0026 (4)	0.0011 (4)	−0.0011 (4)
C9	0.0182 (5)	0.0183 (6)	0.0192 (6)	0.0011 (4)	0.0003 (4)	0.0015 (5)
C10	0.0244 (6)	0.0218 (6)	0.0188 (6)	−0.0007 (5)	0.0002 (5)	0.0054 (5)
C11	0.0210 (6)	0.0258 (6)	0.0141 (5)	−0.0032 (5)	0.0033 (4)	0.0017 (5)
C12	0.0150 (5)	0.0217 (6)	0.0146 (5)	−0.0011 (4)	0.0011 (4)	−0.0027 (4)
C12A	0.0139 (5)	0.0161 (5)	0.0128 (5)	−0.0021 (4)	−0.0010 (4)	−0.0022 (4)
C12B	0.0117 (5)	0.0165 (5)	0.0151 (5)	−0.0014 (4)	0.0012 (4)	−0.0010 (4)
C12C	0.0108 (5)	0.0148 (5)	0.0155 (5)	−0.0007 (4)	0.0018 (4)	−0.0009 (4)
O13	0.0149 (4)	0.0225 (4)	0.0142 (4)	0.0008 (3)	0.0043 (3)	0.0036 (3)
C13	0.0248 (7)	0.0199 (6)	0.0267 (7)	0.0026 (5)	0.0027 (6)	0.0074 (5)
C13'	0.0248 (7)	0.0199 (6)	0.0267 (7)	0.0026 (5)	0.0027 (6)	0.0074 (5)
C14	0.0192 (6)	0.0258 (6)	0.0112 (5)	0.0013 (5)	0.0007 (4)	0.0012 (4)
O14	0.0306 (5)	0.0287 (5)	0.0282 (5)	−0.0025 (4)	−0.0117 (4)	−0.0016 (4)
O15	0.0185 (4)	0.0259 (5)	0.0216 (4)	0.0025 (4)	−0.0020 (3)	0.0031 (4)
C15	0.0177 (6)	0.0360 (7)	0.0217 (6)	0.0042 (5)	−0.0010 (5)	0.0066 (5)
C16	0.0151 (5)	0.0168 (5)	0.0164 (5)	0.0021 (4)	0.0024 (4)	−0.0020 (4)
O16	0.0266 (5)	0.0233 (5)	0.0236 (5)	−0.0069 (4)	−0.0016 (4)	−0.0053 (4)
O17	0.0190 (4)	0.0192 (4)	0.0150 (4)	−0.0035 (3)	0.0011 (3)	0.0015 (3)
C17	0.0274 (6)	0.0215 (6)	0.0246 (6)	−0.0037 (5)	0.0051 (5)	0.0066 (5)
C18	0.0127 (5)	0.0180 (6)	0.0179 (6)	−0.0022 (4)	0.0028 (4)	−0.0001 (4)
C19	0.0243 (6)	0.0231 (6)	0.0167 (6)	−0.0038 (5)	0.0035 (5)	−0.0010 (5)
C20	0.0276 (7)	0.0220 (6)	0.0235 (6)	−0.0045 (5)	0.0069 (5)	−0.0050 (5)
C21	0.0169 (6)	0.0171 (6)	0.0325 (7)	−0.0035 (5)	0.0045 (5)	−0.0018 (5)
C22	0.0218 (6)	0.0198 (6)	0.0250 (6)	−0.0031 (5)	−0.0016 (5)	0.0030 (5)
C23	0.0210 (6)	0.0199 (6)	0.0175 (6)	−0.0025 (5)	0.0010 (4)	−0.0003 (4)
C24	0.0227 (7)	0.0199 (6)	0.0469 (9)	−0.0026 (5)	0.0067 (6)	−0.0021 (6)

### Dimethyl 3-methyl-8-{[4-(trifluoromethyl)phenyl]sulfonyl}-7,8-dihydro-4H-4,6a-epoxybenzo[b]naphtho[1,8-de]azepine-5,6-dicarboxylate Geometric parameters (Å, º)

**Table:**

S1—O1	1.4314 (9)	C10—H10	0.9500
S1—O2	1.4338 (10)	C11—C12	1.3877 (18)
S1—N8	1.6359 (10)	C11—H11	0.9500
S1—C18	1.7642 (13)	C12—C12A	1.4034 (16)
F1—C24	1.3434 (17)	C12—H12	0.9500
F2—C24	1.334 (2)	C12A—C12B	1.4819 (17)
F3—C24	1.343 (2)	C12B—C12C	1.3816 (16)
C1—C2	1.3857 (18)	C13—H13A	0.9800
C1—C12B	1.4129 (17)	C13—H13B	0.9800
C1—H1	0.9500	C13—H13C	0.9800
C2—C3	1.4096 (18)	C13'—H13D	0.9800
C2—H2	0.9500	C13'—H13E	0.9800
C3—C3A	1.3805 (17)	C13'—H13F	0.9800
C3—C13'	1.507 (10)	C14—O14	1.2022 (18)
C3—C13	1.509 (9)	C14—O15	1.3400 (17)
C3A—C12C	1.4001 (17)	O15—C15	1.4562 (15)
C3A—C4	1.5340 (17)	C15—H15A	0.9800
C4—O13	1.4439 (15)	C15—H15B	0.9800
C4—C5	1.5411 (17)	C15—H15C	0.9800
C4—H4	1.0000	C16—O16	1.2045 (16)
C5—C6	1.3416 (18)	C16—O17	1.3452 (15)
C5—C14	1.4820 (17)	O17—C17	1.4378 (15)
C6—C16	1.4795 (17)	C17—H17A	0.9800
C6—C6A	1.5517 (16)	C17—H17B	0.9800
C6A—O13	1.4466 (13)	C17—H17C	0.9800
C6A—C7	1.5198 (16)	C18—C19	1.3876 (17)
C6A—C12C	1.5358 (16)	C18—C23	1.3899 (18)
C7—N8	1.4680 (15)	C19—C20	1.390 (2)
C7—H7A	0.9900	C19—H19	0.9500
C7—H7B	0.9900	C20—C21	1.393 (2)
N8—C8A	1.4405 (15)	C20—H20	0.9500
C8A—C9	1.3928 (17)	C21—C22	1.391 (2)
C8A—C12A	1.4031 (17)	C21—C24	1.4961 (19)
C9—C10	1.3877 (18)	C22—C23	1.3854 (19)
C9—H9	0.9500	C22—H22	0.9500
C10—C11	1.3910 (19)	C23—H23	0.9500
			
O1—S1—O2	121.06 (6)	C12—C12A—C12B	119.67 (11)
O1—S1—N8	106.49 (6)	C12C—C12B—C1	115.67 (11)
O2—S1—N8	107.06 (5)	C12C—C12B—C12A	123.09 (11)
O1—S1—C18	108.27 (6)	C1—C12B—C12A	121.22 (11)
O2—S1—C18	107.08 (6)	C12B—C12C—C3A	122.41 (11)
N8—S1—C18	105.97 (5)	C12B—C12C—C6A	132.85 (11)
C2—C1—C12B	121.64 (11)	C3A—C12C—C6A	104.66 (10)
C2—C1—H1	119.2	C4—O13—C6A	96.88 (8)
C12B—C1—H1	119.2	C3—C13—H13A	109.5
C1—C2—C3	122.36 (11)	C3—C13—H13B	109.5
C1—C2—H2	118.8	H13A—C13—H13B	109.5
C3—C2—H2	118.8	C3—C13—H13C	109.5
C3A—C3—C2	115.46 (11)	H13A—C13—H13C	109.5
C3A—C3—C13'	123.5 (7)	H13B—C13—H13C	109.5
C2—C3—C13'	121.1 (7)	C3—C13'—H13D	109.5
C3A—C3—C13	123.0 (6)	C3—C13'—H13E	109.5
C2—C3—C13	121.5 (6)	H13D—C13'—H13E	109.5
C3—C3A—C12C	122.45 (11)	C3—C13'—H13F	109.5
C3—C3A—C4	133.39 (11)	H13D—C13'—H13F	109.5
C12C—C3A—C4	104.11 (10)	H13E—C13'—H13F	109.5
O13—C4—C3A	100.46 (9)	O14—C14—O15	124.85 (12)
O13—C4—C5	99.91 (10)	O14—C14—C5	126.04 (12)
C3A—C4—C5	105.22 (9)	O15—C14—C5	109.10 (11)
O13—C4—H4	116.3	C14—O15—C15	115.88 (11)
C3A—C4—H4	116.3	O15—C15—H15A	109.5
C5—C4—H4	116.3	O15—C15—H15B	109.5
C6—C5—C14	129.68 (12)	H15A—C15—H15B	109.5
C6—C5—C4	105.54 (11)	O15—C15—H15C	109.5
C14—C5—C4	123.47 (11)	H15A—C15—H15C	109.5
C5—C6—C16	129.54 (11)	H15B—C15—H15C	109.5
C5—C6—C6A	105.26 (10)	O16—C16—O17	124.64 (12)
C16—C6—C6A	122.82 (10)	O16—C16—C6	127.35 (12)
O13—C6A—C7	110.59 (9)	O17—C16—C6	108.01 (10)
O13—C6A—C12C	100.14 (9)	C16—O17—C17	116.09 (10)
C7—C6A—C12C	119.46 (10)	O17—C17—H17A	109.5
O13—C6A—C6	99.57 (9)	O17—C17—H17B	109.5
C7—C6A—C6	119.05 (10)	H17A—C17—H17B	109.5
C12C—C6A—C6	104.71 (9)	O17—C17—H17C	109.5
N8—C7—C6A	113.89 (10)	H17A—C17—H17C	109.5
N8—C7—H7A	108.8	H17B—C17—H17C	109.5
C6A—C7—H7A	108.8	C19—C18—C23	121.92 (12)
N8—C7—H7B	108.8	C19—C18—S1	119.01 (10)
C6A—C7—H7B	108.8	C23—C18—S1	118.95 (10)
H7A—C7—H7B	107.7	C18—C19—C20	119.34 (12)
C8A—N8—C7	118.49 (9)	C18—C19—H19	120.3
C8A—N8—S1	119.23 (8)	C20—C19—H19	120.3
C7—N8—S1	121.75 (8)	C19—C20—C21	118.86 (12)
C9—C8A—C12A	120.98 (11)	C19—C20—H20	120.6
C9—C8A—N8	117.87 (11)	C21—C20—H20	120.6
C12A—C8A—N8	121.12 (10)	C22—C21—C20	121.45 (13)
C10—C9—C8A	120.45 (12)	C22—C21—C24	118.61 (13)
C10—C9—H9	119.8	C20—C21—C24	119.93 (13)
C8A—C9—H9	119.8	C23—C22—C21	119.65 (12)
C9—C10—C11	119.40 (12)	C23—C22—H22	120.2
C9—C10—H10	120.3	C21—C22—H22	120.2
C11—C10—H10	120.3	C22—C23—C18	118.72 (12)
C12—C11—C10	120.20 (12)	C22—C23—H23	120.6
C12—C11—H11	119.9	C18—C23—H23	120.6
C10—C11—H11	119.9	F2—C24—F3	107.37 (13)
C11—C12—C12A	121.37 (11)	F2—C24—F1	106.36 (13)
C11—C12—H12	119.3	F3—C24—F1	105.22 (13)
C12A—C12—H12	119.3	F2—C24—C21	112.84 (13)
C8A—C12A—C12	117.59 (11)	F3—C24—C21	112.32 (13)
C8A—C12A—C12B	122.72 (10)	F1—C24—C21	112.23 (11)
			
C12B—C1—C2—C3	−0.27 (19)	C12—C12A—C12B—C12C	143.46 (12)
C1—C2—C3—C3A	0.97 (18)	C8A—C12A—C12B—C1	146.80 (12)
C1—C2—C3—C13'	−179.4 (8)	C12—C12A—C12B—C1	−34.76 (17)
C1—C2—C3—C13	180.0 (7)	C1—C12B—C12C—C3A	1.32 (17)
C2—C3—C3A—C12C	−0.53 (17)	C12A—C12B—C12C—C3A	−176.99 (11)
C13'—C3—C3A—C12C	179.9 (8)	C1—C12B—C12C—C6A	177.79 (11)
C13—C3—C3A—C12C	−179.5 (7)	C12A—C12B—C12C—C6A	−0.5 (2)
C2—C3—C3A—C4	−177.38 (12)	C3—C3A—C12C—C12B	−0.65 (18)
C13'—C3—C3A—C4	3.0 (8)	C4—C3A—C12C—C12B	177.00 (11)
C13—C3—C3A—C4	3.6 (7)	C3—C3A—C12C—C6A	−177.97 (11)
C3—C3A—C4—O13	−148.68 (13)	C4—C3A—C12C—C6A	−0.32 (11)
C12C—C3A—C4—O13	34.06 (11)	O13—C6A—C12C—C12B	149.70 (12)
C3—C3A—C4—C5	107.93 (15)	C7—C6A—C12C—C12B	28.95 (18)
C12C—C3A—C4—C5	−69.34 (11)	C6—C6A—C12C—C12B	−107.51 (14)
O13—C4—C5—C6	−33.53 (11)	O13—C6A—C12C—C3A	−33.39 (11)
C3A—C4—C5—C6	70.27 (12)	C7—C6A—C12C—C3A	−154.14 (10)
O13—C4—C5—C14	158.45 (10)	C6—C6A—C12C—C3A	69.41 (11)
C3A—C4—C5—C14	−97.75 (13)	C3A—C4—O13—C6A	−54.37 (10)
C14—C5—C6—C16	4.6 (2)	C5—C4—O13—C6A	53.28 (10)
C4—C5—C6—C16	−162.42 (11)	C7—C6A—O13—C4	−179.14 (10)
C14—C5—C6—C6A	167.07 (12)	C12C—C6A—O13—C4	53.93 (10)
C4—C5—C6—C6A	0.07 (12)	C6—C6A—O13—C4	−53.03 (10)
C5—C6—C6A—O13	33.30 (11)	C6—C5—C14—O14	36.1 (2)
C16—C6—C6A—O13	−162.73 (10)	C4—C5—C14—O14	−158.94 (13)
C5—C6—C6A—C7	153.40 (10)	C6—C5—C14—O15	−144.93 (13)
C16—C6—C6A—C7	−42.62 (15)	C4—C5—C14—O15	20.01 (16)
C5—C6—C6A—C12C	−69.93 (11)	O14—C14—O15—C15	−3.06 (18)
C16—C6—C6A—C12C	94.05 (12)	C5—C14—O15—C15	177.98 (10)
O13—C6A—C7—N8	−105.16 (11)	C5—C6—C16—O16	−44.8 (2)
C12C—C6A—C7—N8	10.18 (15)	C6A—C6—C16—O16	155.42 (12)
C6—C6A—C7—N8	140.52 (10)	C5—C6—C16—O17	135.61 (13)
C6A—C7—N8—C8A	−72.75 (13)	C6A—C6—C16—O17	−24.19 (14)
C6A—C7—N8—S1	98.88 (11)	O16—C16—O17—C17	8.00 (17)
O1—S1—N8—C8A	−170.14 (9)	C6—C16—O17—C17	−172.38 (10)
O2—S1—N8—C8A	−39.32 (10)	O1—S1—C18—C19	152.89 (10)
C18—S1—N8—C8A	74.73 (10)	O2—S1—C18—C19	20.83 (11)
O1—S1—N8—C7	18.29 (11)	N8—S1—C18—C19	−93.20 (11)
O2—S1—N8—C7	149.11 (9)	O1—S1—C18—C23	−30.97 (11)
C18—S1—N8—C7	−96.85 (10)	O2—S1—C18—C23	−163.03 (10)
C7—N8—C8A—C9	−107.68 (13)	N8—S1—C18—C23	82.94 (11)
S1—N8—C8A—C9	80.47 (13)	C23—C18—C19—C20	−2.14 (19)
C7—N8—C8A—C12A	70.01 (15)	S1—C18—C19—C20	173.88 (10)
S1—N8—C8A—C12A	−101.83 (12)	C18—C19—C20—C21	0.4 (2)
C12A—C8A—C9—C10	−0.56 (19)	C19—C20—C21—C22	1.6 (2)
N8—C8A—C9—C10	177.14 (11)	C19—C20—C21—C24	−179.74 (12)
C8A—C9—C10—C11	−0.2 (2)	C20—C21—C22—C23	−1.9 (2)
C9—C10—C11—C12	0.9 (2)	C24—C21—C22—C23	179.43 (12)
C10—C11—C12—C12A	−0.7 (2)	C21—C22—C23—C18	0.18 (19)
C9—C8A—C12A—C12	0.70 (17)	C19—C18—C23—C22	1.85 (19)
N8—C8A—C12A—C12	−176.93 (10)	S1—C18—C23—C22	−174.17 (9)
C9—C8A—C12A—C12B	179.17 (11)	C22—C21—C24—F2	−158.19 (13)
N8—C8A—C12A—C12B	1.54 (17)	C20—C21—C24—F2	23.13 (19)
C11—C12—C12A—C8A	−0.05 (18)	C22—C21—C24—F3	−36.65 (18)
C11—C12—C12A—C12B	−178.57 (11)	C20—C21—C24—F3	144.67 (14)
C2—C1—C12B—C12C	−0.87 (17)	C22—C21—C24—F1	81.66 (17)
C2—C1—C12B—C12A	177.47 (11)	C20—C21—C24—F1	−97.02 (16)
C8A—C12A—C12B—C12C	−34.98 (17)		

### Dimethyl 3-methyl-8-{[4-(trifluoromethyl)phenyl]sulfonyl}-7,8-dihydro-4H-4,6a-epoxybenzo[b]naphtho[1,8-de]azepine-5,6-dicarboxylate Hydrogen-bond geometry (Å, º)

**Table:**

*D*—H···*A*	*D*—H	H···*A*	*D*···*A*	*D*—H···*A*
C4—H4···O16^i^	1.00	2.49	3.3893 (16)	149
C7—H7*A*···O1	0.99	2.35	2.8481 (15)	111
C7—H7*B*···O17	0.99	2.46	3.0071 (14)	115
C12—H12···O2^ii^	0.95	2.55	3.1357 (15)	120
C15—H15*A*···O13^ii^	0.98	2.47	3.3741 (17)	153
C17—H17*B*···F1^iii^	0.98	2.54	3.3033 (17)	135
C19—H19···O2	0.95	2.52	2.9003 (17)	104
C22—H22···O14^i^	0.95	2.37	3.2698 (19)	158

Symmetry codes: (i) −*x*+1, *y*−1/2, −*z*+3/2; (ii) *x*+1, *y*, *z*; (iii) *x*+1, *y*+1, *z*.
